# Interactive effect of dietary formic acid and fenugreek on growth performance, immune response, and disease resistance in Nile tilapia challenged with *Aeromonas hydrophila*

**DOI:** 10.1016/j.cirep.2026.200318

**Published:** 2026-09-20

**Authors:** Hussein M.K. Al-Mohammad, Dooha K. Alshamarey, Ahmed J. Obaid, Mahmoud Alagawany, Mohammed F. El Basuini, Akram Ismael Shehata

**Affiliations:** a Al-Furat Al-Awsat Technical University, Diwaniyah, Iraq; b Iraqi Ministry of Education, Diwaniyah, Iraq; c College of Science, Al-Nahrain University, Baghdad, Iraq; d Poultry Department, Faculty of Agriculture, Zagazig University, Zagazig 44511, Egypt; e Animal Production Department, Faculty of Agriculture, Tanta University, Tanta 31527, Egypt; f Department of Animal and Fish Production, Faculty of Agriculture (Saba Basha), Alexandria University, Alexandria 21531, Egypt

**Keywords:** Disease resistance, Formic acid, Gene expression, Growth performance, *Oreochromis niloticus*, *Trigonella foenum-graecum*

## Abstract

This study investigated the interactive effects of dietary formic acid (FA) and fenugreek (FG) supplementation on growth performance, hematological and biochemical parameters, antioxidant status, innate immune response, and disease resistance in Nile tilapia (*Oreochromis niloticus*) challenged with *Aeromonas hydrophila*. A total of 240 juvenile fish (average initial body weight = 21.94 ± 0.04 g) were randomly allocated to four experimental groups following a 2 × 2 factorial design: control (no additive), FA alone (0.5%), FG alone (2%), and a combined FA+FG treatment. Fish received experimental diets for 60 days, after which all groups underwent a standardized bacterial challenge test. Significant improvements in growth were observed across all supplemented groups. Final body weight increased progressively across groups (33.92 g in controls, 43.71 g with FA alone, 47.61 g with FG alone, and 52.84 g with combined FA+FG supplementation; *P* < 0.001), with the combined treatment producing the greatest improvement. Similarly, the FA+FG treatment resulted in the highest specific growth rate (1.46 vs. 0.72%/day in controls) and the most favorable feed conversion ratio (1.37 vs. 1.80 in controls), with significant differences among treatments (*P* < 0.001). Hematological indices (RBC, WBC, Hb, and PCV) and serum biochemical parameters (total protein, albumin, and globulin) were significantly elevated in the treatment groups relative to the control. Antioxidant enzyme activities (SOD and GPx) were markedly increased, while malondialdehyde (MDA) concentrations were significantly reduced, collectively indicating attenuated oxidative stress. Innate immune markers, including lysozyme activity and circulating cytokine levels, were significantly elevated in the supplemented groups. Furthermore, hepatic gene expression analysis revealed significant upregulation of pro-inflammatory cytokines (*IL-1β* and *TNF-α*) and the anti-inflammatory mediator *TGF-β*, suggesting improved immunomodulatory capacity. Following *A. hydrophila* challenge, fish fed the combined FA+FG diet exhibited the highest survival rate. These findings suggest that combined dietary supplementation with formic acid and fenugreek exerts synergistic effects on growth, physiological condition, antioxidant defense, and immune function, ultimately enhancing resistance to bacterial pathogens in Nile tilapia. The results support the potential of this dual-additive strategy as an effective, safe, and sustainable alternative to conventional antibiotic-based therapies in intensive aquaculture.

## Highlights

• Dietary formic acid (0.5% FA) and fenugreek (2% FG) synergistically improved growth performance and feed efficiency in Nile tilapia.
• Combined supplementation significantly enhanced hematological, biochemical, and antioxidant parameters.
• Formic acid and fenugreek elevated lysozyme activity and cytokine-mediated innate immune responses.
• Dietary treatments upregulated hepatic *IL-1β, TNF-α*, and *TGF-β* gene expression, suggesting improved immunomodulatory capacity.
• Combined FA+FG supplementation provided the highest survival and resistance against *Aeromonas hydrophila*.

## Introduction

Aquaculture has emerged as one of the fastest-growing food production sectors globally, playing an indispensable role in ensuring food security and meeting the escalating worldwide demand for high-quality animal protein [1,2]. Among farmed finfish species, Nile tilapia (*Oreochromis niloticus*) occupies a position of exceptional commercial importance, with production systems spanning tropical and subtropical regions across Africa, Asia, and Latin America. Its rapid growth rate, broad environmental tolerance, omnivorous feeding behavior, efficient feed conversion, and consumer acceptance have established it as the world's second most cultured aquaculture species [3,4]. However, the profitability and sustainability of intensive tilapia aquaculture remain constrained by recurrent infectious disease outbreaks, particularly those caused by bacterial pathogens. This represents a critical bottleneck to sector expansion.

The progressive intensification of aquaculture production, however, has been accompanied by an increased incidence of infectious disease outbreaks, which now represent one of the most serious threats to the sustainability and profitability of the sector [5,6]. Stocking densities exceeding 100 fish/m³ in commercial operations create conditions of chronic stress that suppress adaptive immunity, rendering fish highly vulnerable to pathogen invasion [7,8]. Bacterial pathogens are of particular concern; among those affecting tilapia, *Aeromonas hydrophila* is foremost in clinical significance. This Gram-negative, facultatively anaerobic opportunistic pathogen is the primary causative agent of motile aeromonad septicemia (MAS), a systemic hemorrhagic disease characterized by skin ulcerations, abdominal distension, hemorrhagic lesions, and high mortality rates [9]. Alongside *A. hydrophila*, other bacterial pathogens including *Streptococcus agalactiae, Streptococcus iniae, Francisella orientalis*, and *Flavobacterium* spp. have been responsible for recurring mortality events and substantial economic losses in tilapia operations globally [10,11]. The economic and biological toll of these disease challenges is further amplified under intensive production conditions, where the proximity of fish and the rapid transmission of pathogens can devastate entire cohorts within days.

For decades, antibiotics served as the primary tool for managing bacterial disease in aquaculture. However, their widespread use, particularly in Southeast Asia where prophylactic administration remains common, has produced a cascade of well-documented consequences that now severely constrain their continued application. These include the emergence and global dissemination of antimicrobial-resistant bacterial strains, the accumulation of antibiotic residues in fish tissues and surrounding aquatic ecosystems, disruption of non-target microbial communities, and serious downstream implications for human health through the food chain [12,13]. In response, regulatory authorities across numerous countries have imposed increasingly stringent restrictions and in many cases outright bans on antibiotic use in food animal production, creating an urgent and growing demand for effective, safe, and sustainable therapeutic alternatives [14,15]. This regulatory shift has created both a crisis and an opportunity: producers must adopt alternatives, but evidence-based, practical strategies remain limited.

Vaccination represents one such alternative; however, its practical application in aquaculture remains constrained by the high cost of development and production, species-specificity, difficulties with large-scale administration, and the lack of broad-spectrum efficacy against the diverse bacterial threats encountered in farming environments [16]. These limitations have directed considerable scientific attention toward naturally derived bioactive dietary additives including probiotics, prebiotics, phytogenic compounds, and organic acids as viable, antibiotic-free strategies to enhance fish health, stimulate innate immune mechanisms, and improve production performance [17,18]. However, most additive strategies have been evaluated individually; synergistic combinations remain underexplored.

Among these alternatives, organic acids have demonstrated considerable promise as multifunctional feed additives across aquaculture species [19]. Their principal mechanisms of action include reduction of gastrointestinal pH, direct inhibition of pathogenic bacterial proliferation through disruption of microbial cell membranes and intracellular pH homeostasis, enhancement of nutrient digestibility through improved protease activity and mineral absorption, and modulation of beneficial gut microbiota [[20], [21], [22]]. Formic acid (FA), a short-chain monocarboxylic acid, is among the most potent and well-studied organic acids in this context [23]. By acidifying the luminal environment, FA enhances the activity of endogenous proteolytic enzymes such as pepsin, optimizes protein hydrolysis and amino acid absorption, and maintains dietary minerals, particularly iron and phosphorus in their more bioavailable ionic forms, thereby supporting both growth and erythropoiesis [24,25]. Its antimicrobial properties further suppress the proliferation of opportunistic bacteria within the gastrointestinal tract, reducing the energy expenditure associated with subclinical immune activation and channeling more metabolic resources toward somatic growth [[26], [27], [28]]. Dietary FA supplementation has been reported to enhance growth performance, improve hematological and biochemical parameters, and strengthen immune defenses in a range of fish species, including Nile tilapia challenged with *Aeromonas veronii*, where inclusion at 1% significantly elevated white and red blood cell counts, hemoglobin, hematocrit, and serum proteins while reducing post-challenge mortality [29,30].

In parallel, phytogenic feed additives derived from medicinal plants have attracted substantial interest as immunostimulants and growth promoters in aquaculture, owing to their diverse profiles of bioactive phytochemicals and favorable safety and environmental records [31,32]. Unlike organic acids, phytogenic compounds exert their effects predominantly through systemic mechanisms involving absorption of bioactive metabolites, hepatic metabolism, and stimulation of immune cell function. Fenugreek (*Trigonella foenum-graecum* L.), a plant with centuries of culinary and therapeutic history across the Mediterranean region and South Asia, is among the most widely studied of these phytogenic additives in fish nutrition. Its seeds contain a rich array of biologically active constituents including steroidal sapogenins such as diosgenin, the alkaloid trigonelline, 4-hydroxyisoleucine, galactomannans, flavonoids, polyphenols, and saponins that collectively confer antioxidant, anti-inflammatory, antimicrobial, antidiabetic, hypocholesterolemic, and immunomodulatory properties [[33], [34], [35]]. In aquaculture nutrition, dietary supplementation with fenugreek seed powder has been demonstrated to enhance growth performance, improve feed utilization efficiency, modulate hematological and serum biochemical parameters, and augment innate immune responses across multiple teleost species, including Nile tilapia [[36], [37], [38]], gilthead seabream (*Sparus aurata* L.) [39,40], common carp (*Cyprinus carpio* L.) [41], and striped catfish (*Pangasius hypophthalmus*) [42]. The galactomannan fiber component of fenugreek seeds also exhibits a capacity to bind bile acids and reduce intestinal lipid and cholesterol absorption, contributing to improved metabolic efficiency in supplemented animals [34].

Despite the individually documented benefits of both formic acid and fenugreek, their simultaneous application as a combined dietary strategy in aquaculture has not been investigated. This represents a critical gap in the literature with both scientific and practical significance. The two additives operate through mechanistically distinct and potentially complementary pathways: FA at the gastrointestinal interface (local effects) and FG through systemic immune modulation (systemic effects) [43,44]. This mechanistic complementarity raises the specific hypothesis that concurrent supplementation with FA and FG will produce synergistic improvements in growth, antioxidant defense, and immune function by simultaneously reinforcing nutrient bioavailability (FA) and phytochemical absorption (FG), thereby amplifying fenugreek's systemic immunostimulatory effects. Whether such interactions are synergistic or merely additive is a question of both scientific and practical significance, with direct implications for the development of antibiotic-free health management strategies in commercial tilapia aquaculture.

The present study was therefore designed to evaluate, under a controlled 2 × 2 factorial design, the individual and combined effects of dietary formic acid (0.5%) and fenugreek (2%) supplementation on growth performance, hematological and serum biochemical parameters, hepatic antioxidant enzyme activities, innate immune responses, and immune-related gene expression (*IL-1β, TNF-α, TGF-β*) in Nile tilapia. Following the 60-day feeding trial, all fish were subjected to a standardized *A. hydrophila* challenge to assess disease resistance. Specifically, this study aimed to: (i) determine the individual effects of FA and FG on each measured parameter; (ii) determine whether combined FA+FG supplementation produces significant synergistic interactions beyond the effects of either additive alone, as revealed by two-way ANOVA interaction terms; and (iii) evaluate whether these physiological improvements translate to enhanced resistance against bacterial challenge, thereby providing evidence-based guidance for the design of effective, natural, and sustainable dietary health management strategies in intensive Nile tilapia aquaculture.

## Materials and methods

### Ethical statement

All experimental procedures involving Nile tilapia (*Oreochromis niloticus*) were conducted in accordance with international guidelines for the care and use of laboratory animals, including EU Directive 2010/63/EU and the ARRIVE guidelines. The study protocol was reviewed and approved by the Institutional Animal Care and Use Committee (IACUC) of Al-Furat Al-Awsat Technical University under approval number Iq.AAU.REC.2026.024. All efforts were made to minimize animal suffering, reduce the number of fish used, and ensure the reliability and scientific validity of the experimental results.

### Experimental design

This study was designed to investigate the individual and interactive effects of dietary supplementation with formic acid (FA) and fenugreek (FG) on Nile tilapia (*O. niloticus*). A 2 × 2 factorial design was employed, incorporating two dietary factors: formic acid at two levels (0% and 0.5%) and fenugreek at two levels (0% and 2%). The combination of these two factors produced four experimental dietary treatments. The first group (G1) served as the negative control and received the basal diet without any additives. The second group (G2) received the basal diet supplemented with 0.5% formic acid. The third group (G3) received the basal diet supplemented with 2% fenugreek powder. The fourth group (G4) received the basal diet supplemented with both formic acid (0.5%) and fenugreek (2%) simultaneously. Each dietary treatment was randomly assigned to six replicate aquaria, with 10 fish per aquarium, yielding a total of 24 experimental units and 240 fish.

### Experimental fish and husbandry

A total of 240 healthy, mono-sex (all male) juvenile Nile tilapia were obtained from the Aquaculture Research Unit of Al-Furat Al-Awsat Technical University (ATU, Iraq). Mono-sex male fish were selected to minimize growth variance attributable to sex-related differences in anabolic hormone profiles and to ensure experimental uniformity, consistent with common practice in tilapia performance trials [45]. All experimental procedures were conducted in compliance with the Institutional Animal Care and Use Committee (IACUC) guidelines, under approved protocol number 225/2025 (ATU, Iraq). Upon arrival, fish were acclimated for two weeks in aerated holding tanks supplied with dechlorinated water and fed a commercial tilapia diet (ALEKHWA®, Kafrelsheikh, Egypt) at 4% of body mass per day. Following the acclimatization period, fish were individually weighed, and 240 fish with uniform body weights (initial body weight: G1, 21.71 ± 0.12 g; G2, 21.85 ± 0.11 g; G3, 21.93 ± 0.10 g; G4, 22.07 ± 0.09 g; no significant differences among groups, *P* > 0.05) were randomly distributed across 24 glass aquaria (capacity: 100 L each) at a density of 10 fish per aquarium, representing the four dietary treatment groups with six replicates each. The feeding trial was conducted over 60 days under a 12-hour light/dark photoperiod with continuous aeration. Fish were fed the experimental diets twice daily to apparent satiation, with feeding rates initially set at 4% of body mass and adjusted fortnightly in response to changes in tank biomass. Water quality parameters were monitored daily throughout the trial and maintained within the optimal range for Nile tilapia culture: water temperature 26.12 ± 0.26 °C, dissolved oxygen 6.75 ± 0.21 mg/L, pH 7.61 ± 0.24, and total ammonia nitrogen 0.02 ± 0.001 mg/L. Approximately one-third of the tank water volume was exchanged every two days to maintain water quality. Uneaten feed and feces were siphoned daily before the morning feeding.

### Diet formulation and preparation

A basal diet was formulated to meet the nutritional requirements of Nile tilapia as recommended by the National Research Council [46]. The inclusion level of formic acid (0.5%) was selected based on previous studies reporting optimal growth-promoting and gut health benefits in fish at this concentration without adverse effects [29]. Liquid formic acid (98% purity) was diluted with distilled water and uniformly sprayed onto the mixed basal ingredients during pelleting. The added moisture was compensated by reducing the amount of water used during dough preparation, and the pellets were subsequently dried at 40 °C to achieve a final moisture content of approximately 10%. Fenugreek (*Trigonella foenum-graecum*) seeds were oven-dried at 40 °C, ground into a fine powder using a laboratory mill, and incorporated into the respective diets at an inclusion level of 2%, which has previously been demonstrated to exert optimal immunomodulatory and growth-enhancing effects in Nile tilapia without negative impacts on feed palatability or fish health [47]. In the treatment diets (G2, G3, and G4), formic acid and/or fenugreek powder were incorporated into the formulation by proportionally replacing an equivalent weight of wheat flour, thereby maintaining the total diet composition at 100% across all groups. All dry dietary ingredients were thoroughly weighed, blended using a mechanical mixer, moistened with water to facilitate pellet formation, and then pelleted using a laboratory pellet mill. The resulting pellets were air-dried at room temperature and stored in sealed polyethylene bags at 4 °C until use. The ingredient composition and proximate analysis of the four experimental diets are presented in Table 1.

**Table 1:** Ingredient composition and proximate analysis (%) of the four experimental diets used in the feeding trial.

Ingredient (%)	G1 Control	G2 (0.5% FA)	G3 (2% FG)	G4 (FA+FG)
Soybean meal (44% CP)	40.00	40.00	40.00	40.00
Fish meal (65% CP)	5.00	5.00	5.00	5.00
Corn gluten meal (60% CP)	10.00	10.00	10.00	10.00
Yellow corn	10.00	10.00	10.00	10.00
Rice bran	10.00	10.00	10.00	10.00
Wheat bran	12.00	12.00	12.00	12.00
Fish oil	1.00	1.00	1.00	1.00
Soy oil	1.00	1.00	1.00	1.00
Polysorbate 60	3.00	3.00	3.00	3.00
Dicalcium phosphate (DCP)	1.00	1.00	1.00	1.00
Vitamin–mineral premix	0.30	0.30	0.30	0.30
Choline chloride	0.05	0.05	0.05	0.05
Vitamin C (STAY-C 35) 1	0.05	0.05	0.05	0.05
Wheat flour (filler) 2	6.60	6.10	4.60	4.10
Fenugreek powder (FG) 3	0.00	0.00	2.00	2.00
Formic acid (FA liquid, 98%) 4	0.00	0.50	0.00	0.50
**Total**	**100.00**	**100.00**	**100.00**	**100.00**
Proximate composition (%)				
Crude protein	30.1	30.0	30.2	30.1
Crude lipid	6.0	6.0	6.0	6.0
Ash	6.1	6.1	6.2	6.2
Crude fiber	6.8	6.8	6.9	6.9
Moisture	10.0	10.0	10.0	10.0
Gross energy (MJ/kg) 5	18.2	18.2	18.2	18.2

1 STAY-C 35: stabilized ascorbyl polyphosphate (35% active vitamin C).

2 Wheat flour was used as a filler to compensate for the inclusion of fenugreek and formic acid, ensuring that all diets remained isonitrogenous, isolipidic, and isoenergetic.

3 Fenugreek powder composition (analyzed): Moisture 8.2%, crude protein 28.5%, crude lipid 6.8%, crude fiber 6.2%, ash 3.5%, and total phenolic content 12.4 mg gallic acid equivalents (GAE)/g.

4 Formic acid: Liquid formic acid (98% purity) was dissolved in distilled water and sprayed evenly onto the mixed basal ingredients during pelleting. The additional moisture was compensated by reducing the amount of water added to the dough, and the pellets were dried at 40 °C to restore the final moisture content to 10%.

5 Gross energy was calculated using the following conversion factors: protein 23.6 kJ/g, lipid 39.5 kJ/g, and carbohydrate 17.2 kJ/g.

### Growth performance evaluation

All fish in each aquarium were individually weighed at the beginning and end of the 60-day feeding trial following 24-hour fasting. Growth performance indices were calculated using the following standard equations:

$$\text{Weight}\;\text{Gain}\;\left(\text{WG},\;g\right)=W_{60}-W_{0}$$

$$\text{Specific}\;\text{growth}\;\text{rate}\;\left(\text{SGR},\;\%/\text{day}\right)=\frac{\text{Ln}\;W_{60\;\text{day}}-\text{Ln}\;W_{O\;\text{day}}}{t}\times 100$$

$$\text{Feed}\;\text{conversion}\;\text{ratio}\;\left(\text{FCR}\right)=\frac{\text{Total}\;\text{dry}\;\text{feed}\;\text{consumed}\;\left(g\right)}{\text{Total}\;\text{weight}\;\text{gain}\;\left(g\right)}$$

$$\text{Survival}\;\text{rate}\;\left(\text{SR},\;\%\right)=\frac{\text{Number}\;\text{of}\;\text{fish}\;\text{surviving}}{\text{Initial}\;\text{number}\;\text{of}\;\text{fish}}\times 100$$

where W_60_ is the final mean body weight (g), *W₀* is the initial mean body weight (g), and t is the duration of the feeding trial in days.

### Blood sampling and hematological analysis

At the conclusion of the 60-day feeding trial, blood samples were collected from six randomly selected fish per treatment. Before blood collection, fish were fasted for 24 h and then anaesthetized using tricaine methanesulfonate (MS-222) at a concentration of 100 mg/L. Blood was withdrawn from the caudal vein using heparinized syringes. Each blood sample was divided into two portions: one portion was transferred into EDTA-anticoagulated tubes for immediate hematological analysis, and the second portion was allowed to clot at room temperature, then centrifuged at 3000 rpm for 10 min at 4°C to obtain serum for biochemical analyses.

Hematological indices, including red blood cell count (RBC, ×10⁶/mm³), white blood cell count (WBC, ×10³/mm³), and differential leukocyte count, were determined using a hemocytometer. Hemoglobin (Hb) concentration was measured using the cyanmethemoglobin method, and packed cell volume (PCV, hematocrit) was determined by the microhematocrit centrifugation technique. All hematological procedures followed the standard fish hematology methods described by Blaxhall [48]. Serum biochemical parameters, including total protein concentration at 562 nm (Cat. No. A045-3) and albumin concentration at 628 nm (Cat. No. A028-1-1), were quantified using commercial diagnostic kits obtained from Nanjing Jiancheng Bioengineering Institute (Jiangsu, China) according to the manufacturer’s instructions. Serum globulin concentration was calculated as the difference between total protein and albumin values.

### Assessment of innate immune status and oxidative stress biomarkers

Innate immune status and oxidative stress biomarkers were evaluated using serum and hepatic tissue samples. Serum lysozyme activity (LYZ; Cat. No. A050-1-1), interleukin-1 beta (IL-1β; Cat. No. H002-1-1), tumor necrosis factor-alpha (TNF-α; Cat. No. H052-1-1), and transforming growth factor-beta (TGF-β; Cat. No. H034-1-1) concentrations were quantified using commercial diagnostic kits according to the manufacturer’s instructions. For oxidative stress assessment, hepatic tissues were excised, weighed, and homogenized in ice-cold physiological saline (1:9, w/v). The homogenates were centrifuged at 3000 rpm for 15 min at 4 °C, and the collected supernatants were used for antioxidant analyses. Superoxide dismutase (SOD) activity was measured using the WST-1 reduction method at 450 nm (Cat. No. A001-3-2). Glutathione peroxidase (GPx) activity was determined using a colorimetric method at 412 nm (Cat. No. A005-1). Malondialdehyde (MDA) concentration was measured using the thiobarbituric acid reactive substances (TBARS) assay at 532 nm (Cat. No. A003-1). All biomarkers were analyzed using commercial diagnostic kits obtained from Nanjing Jiancheng Bioengineering Institute (Nanjing, China) according to the manufacturer’s instructions.

### Gene expression analysis

At the end of the challenge period, liver tissue samples were collected from six fish per treatment group, immediately immersed in RNAlater (Sigma-Aldrich, USA), and maintained at 4 °C for 24 h before storage at −80 °C until processing. Total RNA was extracted from each liver sample using the ABT RNA Purification Kit (Applied Biotechnology, Cairo, Egypt) according to the manufacturer's instructions. RNA concentration and purity were assessed spectrophotometrically using a NanoDrop spectrophotometer (BioDrop, Cambridge, UK); samples with an A260/A280 ratio of 1.8–2.0 were considered acceptable. All RNA samples were diluted to a uniform concentration of 50 ng/μL before complementary DNA (cDNA) synthesis.

First-strand cDNA was synthesized from total RNA by reverse transcription at 50°C for 30 min using a commercial RT kit. Quantitative real-time PCR (RT-qPCR) was performed using SYBR Green chemistry to evaluate the hepatic expression of the following immune-related target genes: interleukin-1 beta (*IL-1β*), tumor necrosis factor-alpha (*TNF-α*), and transforming growth factor-beta (*TGF-β*). Gene-specific primer sequences are provided in Table 2. The thermocycling program consisted of an initial denaturation step at 95 °C for 10 min, followed by 45 amplification cycles of denaturation at 95 °C for 5 s and combined annealing/extension at 60 °C for 30 s. Amplification specificity was confirmed by melt curve analysis at the end of each run. Beta-actin (*β-actin*) was used as the reference housekeeping gene. Prior to relative quantification analysis, *β-actin* expression stability was evaluated across all treatment groups using the geNorm algorithm (implemented in qbase+ software) and the BestKeeper tool. The calculated M-value was 0.38 (below the threshold of 0.5), and the coefficient of variation (CV) for cycle threshold (Ct) values was <2.5%, confirming stable expression and suitability as a reference gene for RT-qPCR normalization. Relative gene expression levels were calculated using the 2^−ΔΔCt^ method [49], with the control group (G1) serving as the calibrator.

**Table 2:** Primer gene-specific sequences used for quantitative real-time PCR analysis.

Gene	Primer direction	Primer sequence (5′–3′)	Tm ( °C)	Amplicon size (bp)	Efficiency (%)	Accession No.
*IL-1β*	Forward	GCTGAAGAGACGGAAGATGG	60	168	98.5	KF747686.1
	Reverse	CGACTTCTCTGCCTTTCTACC				
*TNF-α*	Forward	CAGCAGTTCGTGTCCTGTGA	60	145	97.2	AY428948.1
	Reverse	GTCGTCAAGCACAGGACACT				
*TGF-β*	Forward	GTTGGATGTGAGCCTCGTAG	58	160	99.0	XM_005477191.4
	Reverse	CAACCTACACTCGCAGCATC				
*β-actin*	Forward	CAGATCGTG GGCGACATACA	60	140	100.2	EU887951.1
	Reverse	GTCTAGCACCCGCTGTATGT				

*IL-1β*: interleukin-1 beta; *TNF-α*: tumor necrosis factor-alpha; *TGF-β*: transforming growth factor-beta.

### Bacterial challenge with aeromonas hydrophila

The *A. hydrophila* strain (GenBank Accession No. LC012339) was maintained on tryptic soy agar (TSA) at 37 °C for 24 h prior to challenge. Single colonies were picked, transferred into tryptic soy broth (TSB), and incubated with constant agitation at 150 rpm and 37 °C for 18 h to reach mid-exponential phase. Bacterial concentration was enumerated by serial tenfold dilution and spread-plating onto TSA, with colony counts performed after 24 h of incubation at 37 °C using the drop plate enumeration method. An initial dose-response trial was conducted to determine the LD_50_ of *A. hydrophila*. A total of 60 fish (10 per dose) were injected intraperitoneally with 0.2 mL of bacterial suspensions at concentrations of 1 × 10⁵, 5 × 10⁵, 1 × 10⁶, 5 × 10⁶, 1 × 10⁷, and 1 × 10⁸ CFU/mL. Fish were observed for 14 days, and mortality was recorded daily. The LD_50_ (2.7 × 10⁶ CFU/mL, equivalent to 5.4 × 10⁵ CFU/fish) was calculated using probit analysis. This initial trial was conducted under the same ethical approval (Iq.AAU.REC.2026.024), and the fish were humanely euthanized at the end of the observation period. The working bacterial suspension was prepared in sterile phosphate-buffered saline (PBS, pH 7.4) at a final concentration of 2.7 × 10⁶ CFU/mL (equivalent to 5.4 × 10⁵ CFU/fish in a 0.2 mL injection volume), corresponding to the experimentally determined LD_50_.

At the conclusion of the 60-day feeding trial, fish in all experimental groups were allowed a 24-hour rest period following blood sampling to minimize sampling-induced physiological stress. Each fish was then intraperitoneally injected with 0.2 mL of the *A. hydrophila* suspension at the established LD_50_ concentration. Fish were maintained in their respective aquaria under standard husbandry conditions for 14 days post-challenge. Mortalities were recorded daily, and dead fish were promptly removed and subjected to post-mortem bacterial re-isolation and confirmation on TSA to verify the cause of death. The cumulative survival rate (%) was calculated at the end of the 14-day observation period.

### Statistical analysis

All data were expressed as mean ± standard error (SE). Prior to analysis, data normality was confirmed using the Shapiro-Wilk test and homogeneity of variance was verified using Levene's test; both assumptions were satisfied for all variables (*P* > 0.05). Statistical analyses were then performed using IBM SPSS Statistics version 23.0 (SPSS Inc., Chicago, IL, USA). Differences among the experimental groups were analyzed using a two-way analysis of variance (ANOVA) to evaluate the main effects of dietary formic acid (FA), fenugreek (FG), and their interaction (FA × FG). When significant differences were detected, Tukey’s honestly significant difference (HSD) post hoc test was used for pairwise comparisons of treatment means. Significant pairwise comparisons (Tukey’s HSD) and two-way ANOVA results are presented in the corresponding tables. Survival data following the challenge test were additionally analyzed using the Chi-square (X^2^) test. Statistical significance was considered at *P* < 0.05.

## Results

### Growth performance and feed utilization

Dietary supplementation with formic acid (FA), fenugreek (FG), and their combination significantly improved the growth performance, feed utilization, and survival of Nile tilapia compared with the control group (Table 3). Fish fed the combined FA+FG diet (G4) exhibited the highest final body weight, weight gain, and specific growth rate, followed by the FG group (G3), FA group (G2), and control group (G1) (*P* = 0.001; all pairwise comparisons significant). Feed conversion ratio (FCR) was significantly improved in all supplemented groups, with the best feed efficiency observed in G4 (*P* = 0.001). No mortality occurred during the 60-day feeding period; all groups demonstrated 100% survival. Two-way ANOVA further demonstrated significant main effects of formic acid (FA) and fenugreek (FG), as well as significant FA × FG interactions, on final weight, weight gain, specific growth rate (SGR), and feed conversion ratio (FCR) (*P* = 0.001). The significant interaction indicates that the effect of FA on growth performance depended on the presence of FG, with the combined FA+FG treatment producing the greatest improvement among the experimental groups.

**Table 3:** Effects of dietary formic acid and fenugreek supplementation on growth performance, feed utilization, and survival of Nile tilapia (*Oreochromis niloticus*) after 60 days of feeding (n = 6 per group).

Variables	G1 Control	G2 (0.5% FA)	G3 (2% FG)	G4 (FA+FG)	Two-Way ANOVA		
					FA	FG	FA×FG
Initial weight, g	21.91 ± 0.06	21.95 ± 0.06	22.01 ± 0.07	22.07 ± 0.09	0.409	0.087	0.865
Final weight, g	33.92 ± 0.10 ^d^	43.71 ± 0.13 ^c^	47.61 ± 0.09 ^b^	52.84 ± 0.16 ^a^	0.001	0.001	0.001
Weight gain, g	12.00 ± 0.10 ^d^	21.75 ± 0.14 ^c^	25.60 ± 0.12 ^b^	30.77 ± 0.13 ^a^	0.001	0.001	0.001
SGR, %/day	0.72 ± 0.01 ^d^	1.15 ± 0.01 ^c^	1.29 ± 0.01 ^b^	1.46 ± 0.01 ^a^	0.001	0.001	0.001
FCR	1.80 ± 0.06 ^a^	1.64 ± 0.02 ^b^	1.43 ± 0.02 ^c^	1.37 ± 0.02 ^d^	0.001	0.001	0.001
Survival, %	100	100	100	100	n/a	n/a	n/a

The values are represented as mean ± SE. The different letters within a row indicate significant differences at *P* < 0.05. *P*-values represent the main effects of formic acid (FA) and fenugreek (FG), and their interaction (FA × FG), as determined by two-way ANOVA. SGR, specific growth rate; FCR, feed conversion ratio; n/a., not significant.

### Hematological and biochemical parameters

Dietary supplementation with formic acid (FA), fenugreek (FG), and their combination significantly improved the hematological and serum biochemical parameters of Nile tilapia compared with the control group (Table 4). Fish fed the combined FA+FG diet (G4) exhibited the highest values of red blood cells (RBC), white blood cells (WBC), hemoglobin (Hb), packed cell volume (PCV), total protein, albumin, and globulin levels, followed by the FG group (G3), FA group (G2), and control group (G1) (*P* = 0.001; all pairwise comparisons significant). Two-way ANOVA revealed significant main effects of formic acid (FA) and fenugreek (FG) on all evaluated hematological and biochemical parameters (*P* = 0.001). Significant FA × FG interactions were also detected for RBC, WBC, total protein, albumin, and globulin, indicating that the effects of FA and FG on these parameters were interdependent. In contrast, no significant FA × FG interaction was detected for hemoglobin concentration (Hb) (*P* = 0.295), indicating independent effects of the two dietary additives on this parameter.

**Table 4:** Effects of dietary formic acid and fenugreek supplementation on hematological and serum biochemical parameters of Nile tilapia (*Oreochromis niloticus*) after 60 days of feeding (n = 6 per group).

Variables	G1 Control	G2 (0.5% FA)	G3 (2% FG)	G4 (FA+FG)	Two-Way ANOVA		
					FA	FG	FA×FG
RBC (10^6^/mm^3^)	1.70 ± 0.02 ^d^	2.06 ± 0.01 ^c^	2.33 ± 0.04 ^b^	2.89 ± 0.01 ^a^	0.001	0.001	0.001
WBC (10^3^/mm^3^)	44.77 ± 0.16 ^d^	49.57 ± 0.16 ^c^	51.20 ± 0.10 ^b^	53.40 ± 0.11 ^a^	0.001	0.001	0.001
Hb (g/dl)	6.19 ± 0.01 ^c^	7.24 ± 0.04 ^b^	7.24 ± 0.01 ^b^	8.35 ± 0.08 ^a^	0.001	0.001	0.295
PCV, %	25.27 ± 0.02 ^c^	26.05 ± 0.03 ^b^	26.85 ± 0.04 ^a^	27.8 ± 0.3 ^a^	0.001	0.001	0.001
Total protein (g/dl)	3.34 ± 0.06 ^d^	4.01 ± 0.01 ^c^	4.44 ± 0.02 ^b^	5.49 ± 0.04 ^a^	0.001	0.001	0.001
Albumin (g/dl)	1.37 ± 0.01 ^d^	1.69 ± 0.01 ^c^	1.89 ± 0.00 ^b^	2.26 ± 0.01 ^a^	0.001	0.001	0.003
Globulin (g/dl)	1.42 ± 0.02 ^d^	1.88 ± 0.01 ^c^	2.19 ± 0.02 ^b^	3.02 ± 0.04 ^a^	0.001	0.001	0.001

The values are represented as mean ± SE. The different letters within a row indicate significant differences at *P* < 0.05. *P*-values represent the main effects of formic acid (FA) and fenugreek (FG), and their interaction (FA × FG), as determined by two-way ANOVA. RBC, red blood cells; WBC, white blood cells; Hb, hemoglobin; PCV, packed cell volume.

### Antioxidant status and immune parameters

Dietary supplementation with formic acid (FA), fenugreek (FG), and their combination significantly enhanced the antioxidant status and immune responses of Nile tilapia compared with the control group (Fig. 1, Fig. 2). Fish fed the combined FA+FG diet (G4) exhibited the highest activities of superoxide dismutase (SOD) and glutathione peroxidase (GPx), along with the lowest malondialdehyde (MDA) levels, indicating enhanced antioxidant defense and reduced lipid peroxidation (*P* = 0.001; all pairwise comparisons significant) (Fig. 1). Two-way ANOVA revealed significant main effects of FG on SOD activity (*P* = 0.001), whereas the effects of FA and the FA × FG interaction were not significant (*P* = 0.280 and 0.567, respectively). GPx activity and MDA levels were significantly influenced by FA, FG, and their interaction (*P* ≤ 0.004).

**Fig. 1:**
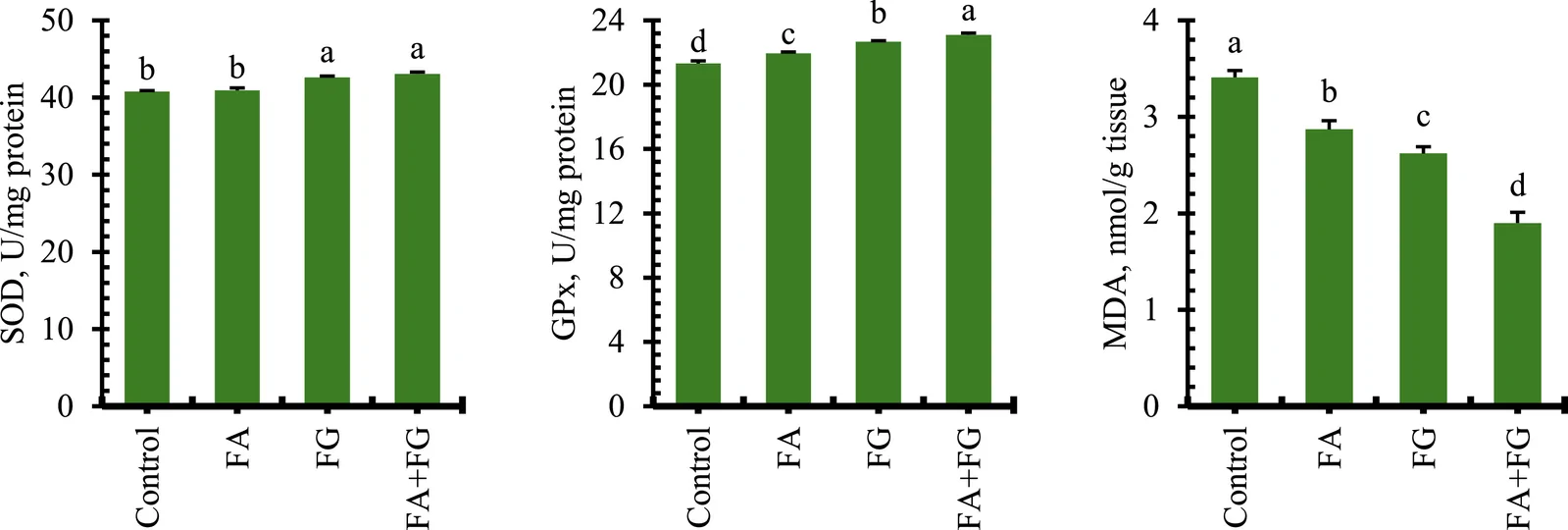
Effects of dietary formic acid and fenugreek supplementation on SOD: superoxide dismutase (*P*-Values: FA= 0.280; FG= 0.001; FA×FG=0.567), GPx: glutathione peroxidase (*P*-Values: FA= 0.001; FG= 0.001; FA×FG=0.004), MDA: malondialdehyde (*P*-Values: FA= 0.001; FG= 0.001; FA×FG=0.001) in Nile tilapia (*Oreochromis niloticus*) after 60 days of feeding (n = 6 per group).

**Fig. 2:**
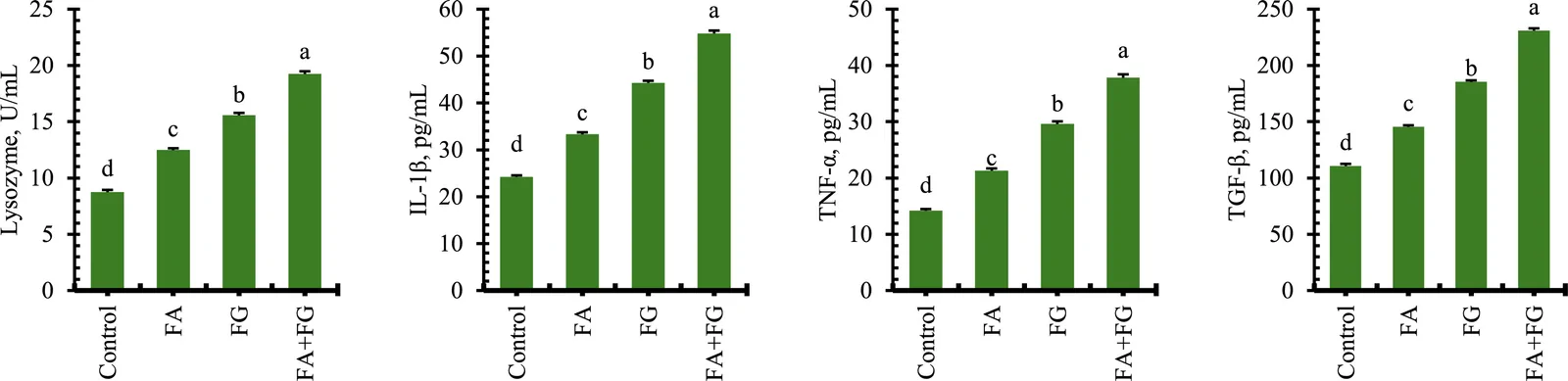
Effects of dietary formic acid and fenugreek supplementation on lysozyme (*P*-Values: FA= 0.001; FG= 0.001; FA×FG=0.629), IL-1β (*P*-Values: FA= 0.001; FG= 0.001; FA×FG=0.004), TNF-α (*P*-Values: FA= 0.001; FG= 0.001; FA×FG=0.001), and TGF-β (*P*-Values: FA= 0.001; FG= 0.001; FA×FG=0.629) in Nile tilapia (*Oreochromis niloticus*) after 60 days of feeding (n = 6 per group). IL-1β: interleukin-1 beta; TNF-α: tumor necrosis factor-alpha; TGF-β: transforming growth factor-beta.

Moreover, lysozyme activity and immune-related cytokines, including IL-1β, TNF-α, and TGF-β, were significantly elevated in all supplemented groups, with the greatest enhancement observed in G4 (Fig. 2). Significant main effects of FA and FG were also observed for lysozyme, IL-1β, TNF-α, and TGF-β levels (*P* = 0.001). However, no significant FA × FG interaction effect was detected for lysozyme and TGF-β (*P* = 0.629), while significant interaction effects were recorded for IL-1β and TNF-α (*P* = 0.001).

### Immune-related gene expression

Dietary supplementation with formic acid (FA), fenugreek (FG), and their combination significantly upregulated the expression levels of immune-related genes in Nile tilapia compared with the control group (Table 5). Fish fed the combined FA+FG diet (G4) exhibited the highest expression levels of *IL-1β, TNF-α*, and *TGF-β*, followed by the FG group (G3), FA group (G2), and control group (G1) (*P* = 0.001; all pairwise comparisons significant). Two-way ANOVA revealed significant main effects of both FA and FG on the expression levels of *IL-1β, TNF-α*, and *TGF-β* (*P* = 0.001). Significant FA × FG interaction effects were also observed for *IL-1β* (*P* = 0.001) and *TNF-α* (*P* = 0.024), whereas no significant interaction effect was detected for *TGF-β* expression (*P* = 0.522).

**Table 5:** Effects of dietary formic acid and fenugreek supplementation on immune-related gene expression of Nile tilapia (*Oreochromis niloticus*) after 60 days of feeding (n = 6 per group).

Variables	G1 Control	G2 (0.5% FA)	G3 (2% FG)	G4 (FA+FG)	Two-Way ANOVA		
					FA	FG	FA×FG
*IL-1β*	25.72 ± 0.26 ^d^	29.14 ± 0.10 ^c^	31.88 ± 0.14 ^b^	33.87 ± 0.11 ^a^	0.001	0.001	0.001
*TNF-α*	14.67 ± 0.10 ^d^	16.35 ± 0.06 ^c^	18.52 ± 0.25 ^b^	20.88 ± 0.45 ^a^	0.001	0.001	0.024
*TGF-β*	112.05 ± 0.38 ^c^	115.68 ± 0.40 ^b^	121.30 ± 1.40 ^a^	124.18 ± 0.18 ^a^	0.001	0.001	0.522

The values are represented as mean ± SE. The different letters within a column indicate significant differences at *P* < 0.05. *P*-values represent the main effects of formic acid (FA) and fenugreek (FG), and their interaction (FA × FG), as determined by two-way ANOVA. *IL-1β*, interleukin-1 beta; *TNF-α*, tumor necrosis factor-alpha; *TGF-β*, transforming growth factor-beta.

### Aeromonas hydrophila challenge

Following the *Aeromonas hydrophila* challenge, dietary supplementation with formic acid (FA), fenugreek (FG), or their combination significantly improved Nile tilapia survival compared with the control group (Fig. 3). Fish fed the combined FA+FG diet (G4) exhibited the highest survival and lowest mortality, followed by the FG group (G3) and FA group (G2), whereas the control group (G1) showed the lowest resistance to bacterial challenge. The Chi-square analysis confirmed significant differences in survival distribution among the experimental groups, indicating enhanced disease resistance in fish receiving dietary supplementation, particularly the combined treatment.

**Fig. 3:**
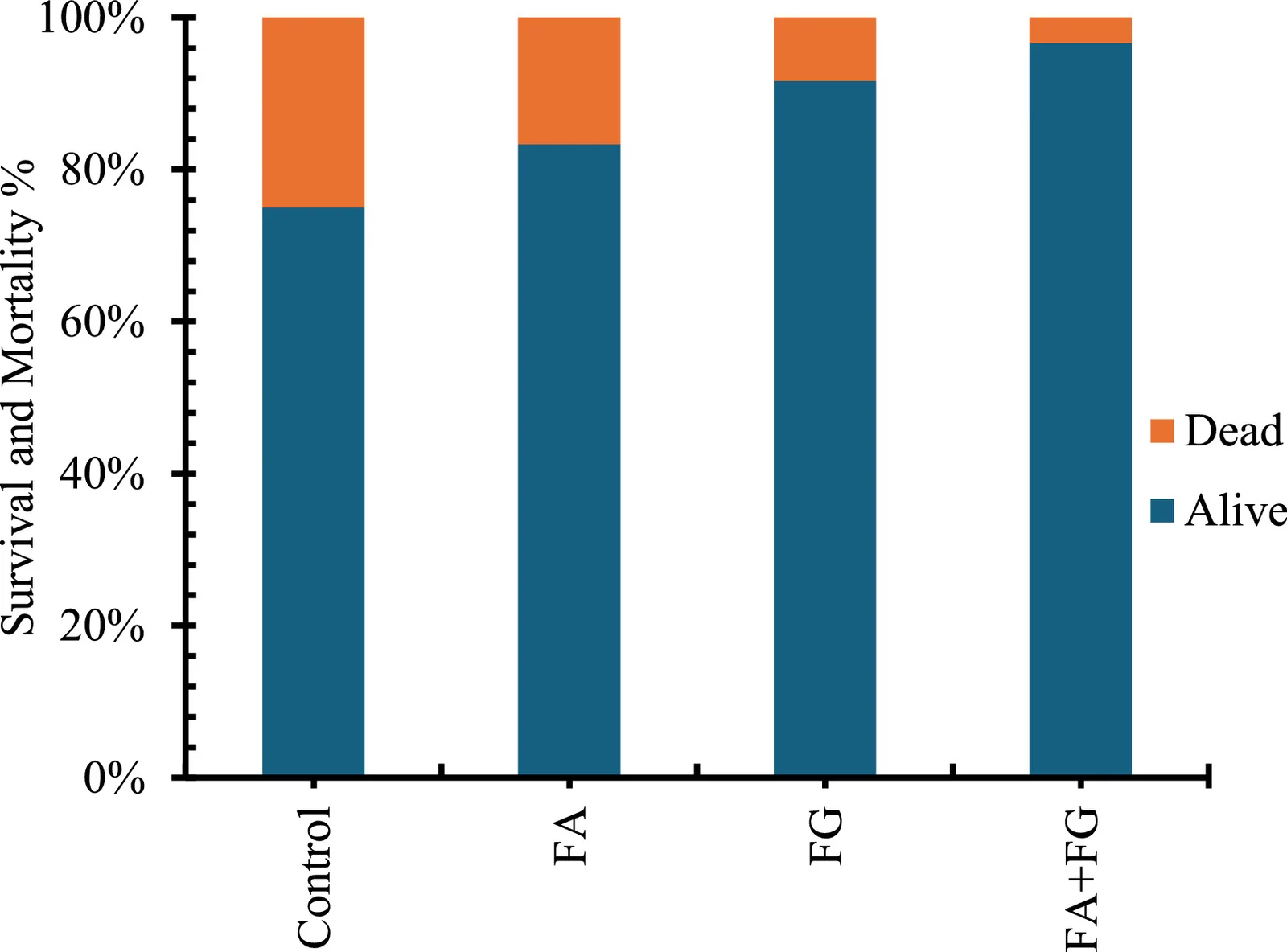
Survival of Nile tilapia (*Oreochromis niloticus*) following experimental challenge with *Aeromonas hydrophila* after 60 days of dietary supplementation with formic acid (FA), fenugreek (FG), or their combination (FA+FG). Fish were monitored for 14 days after intraperitoneal challenge. Bars represent the percentage of surviving and dead fish in each treatment group (n = 60 fish per group; 10 fish × 6 replicates). Survival differed significantly among treatments (Pearson χ² = 14.135, df = 3, *P* = 0.003). FA, formic acid; FG, fenugreek.

## Discussion

The present study investigated the individual and combined effects of dietary formic acid (FA) and fenugreek (FG) supplementation on growth performance, hematological and biochemical parameters, antioxidant defense, innate immune responses, immune-related gene expression, and resistance to *A. hydrophila* challenge in Nile tilapia (*O. niloticus*). The results consistently demonstrated that both additives improved the measured health and performance parameters, with the combined FA+FG treatment (G4) producing the most pronounced effects across virtually all variables. Two-way ANOVA revealed significant synergistic interactions between FA and FG across most parameters examined, underscoring the potential of this dual-additive strategy as a viable, antibiotic-free alternative in sustainable Nile tilapia aquaculture.

Dietary supplementation with formic acid and fenugreek significantly enhanced growth performance in Nile tilapia, with the combined FA+FG treatment yielding the highest final body weight, weight gain, and specific growth rate (SGR), alongside the lowest (most favorable) feed conversion ratio (FCR). These findings are consistent with the well-established mechanisms by which organic acids promote fish growth. Formic acid, as a short-chain monocarboxylic acid, lowers luminal pH in the gastrointestinal tract, thereby enhancing the activity of endogenous proteolytic enzymes such as pepsin, optimizing protein hydrolysis, and improving the absorption of amino acids and minerals, particularly iron and phosphorus, that are essential for metabolic and anabolic processes [24,50]. In addition, the antimicrobial properties of formic acid suppress the proliferation of pathogenic and opportunistic bacteria within the gut, reducing the metabolic energy expenditure associated with immune activation and directing more dietary energy toward somatic growth [[26], [27], [28]]. Similar improvements in growth performance and feed efficiency following dietary organic acid supplementation have been reported in several fish species, including Nile tilapia (*Oreochromis niloticus*) [29,51], Beluga (*Huso*) [52], common carp (*Cyprinus carpio*) [53,54], and largemouth bass (*Micropterus salmoides*) [28], where formic acid and its salts enhanced nutrient utilization, intestinal health, and growth indices.

The growth-promoting effects of fenugreek are similarly well documented and are attributable to its diverse profile of bioactive phytochemicals. Saponins and flavonoids present in fenugreek seeds enhance feed palatability, stimulate the secretion of digestive enzymes, and improve intestinal morphology, notably by increasing villus height, villus surface area, and the villus height-to-crypt depth ratio, thereby maximizing the absorptive capacity of the intestinal epithelium [47,55]. The flavonoid constituents of fenugreek also exert potent antioxidant effects, reducing the formation of reactive oxygen species (ROS) in intestinal tissue, protecting mucosal integrity and further contributing to efficient nutrient absorption [34,56]. These mechanisms collectively translate into improved feed utilization efficiency and enhanced growth, as evidenced by the significantly lower FCR in the FG group compared with the control. Similar beneficial effects of dietary fenugreek supplementation on growth performance and feed utilization have been reported in Nile tilapia (*Oreochromis niloticus*) [38], gilthead seabream (*Sparus aurata* L.) [39,57], common carp (*Cyprinus carpio*) [41], and striped catfish (*Pangasius hypophthalmus*) [42]. These findings support the improved growth and feed efficiency observed in the present study, particularly in fish receiving the combined FA+FG supplementation.

Of particular significance, two-way ANOVA identified significant synergistic interactions between FA and FG on final body weight, weight gain, and SGR (*P* < 0.001), indicating that the growth-promoting effects of the combined treatment exceeded the sum of the individual contributions of each additive. One possible, although untested, explanation is that formic acid lowers the pH of the gastrointestinal tract, which may increase the solubility and bioavailability of the bioactive compounds in fenugreek. This may enhance their digestive and metabolic effects. However, this mechanism was not examined in the present study. Future research should investigate it by measuring intestinal pH and the absorption of fenugreek bioactive compounds [[57], [58], [59]]. This pattern is consistent with the broader principle in functional feed supplementation research, in which multi-component formulations frequently outperform single-additive strategies by simultaneously targeting complementary physiological pathways [54,60,61]. Taken together, these findings suggest that combined dietary supplementation with formic acid and fenugreek represents a promising nutritional strategy to optimize growth performance and feed utilization in intensive Nile tilapia culture systems.

Hematological indices are widely employed as sensitive and informative indicators of fish health, physiological status, and immune competence, reflecting the integrated effects of nutrition, stress, and pathogen exposure on erythropoietic and leukocytic function [62]. In the present study, dietary supplementation with FA, FG, or their combination significantly elevated RBC and WBC counts, hemoglobin (Hb) concentration, and packed cell volume (PCV), with the greatest improvements observed in the combined treatment group (G4).

The significant increases in RBC count, Hb concentration, and PCV in the supplemented groups are indicative of enhanced erythropoietic activity and improved oxygen-carrying capacity of the blood, supporting heightened aerobic metabolism, tissue oxygenation, and growth [63]. These improvements are mechanistically linked to the capacity of organic acids to enhance the intestinal absorption of erythropoietic minerals, particularly iron, through luminal acidification, which maintains iron in its more soluble ferrous (Fe²⁺) form and thereby promotes hemoglobin biosynthesis [24,64]. The observation that fenugreek supplementation also significantly elevated erythropoietic indices is consistent with previous studies in gilthead seabream (*S. aurata*) and Nile tilapia, where fenugreek-fed fish exhibited significantly higher RBC counts and Hb concentrations than controls [38,39]. This effect is attributed to the antioxidant flavonoids and phenolic compounds in fenugreek, which reduce oxidative damage to erythrocyte membranes, thereby prolonging erythrocyte survival and improving the overall red blood cell profile [65,66]. Importantly, the dose-dependent enhancement in RBC count, Hb, and PCV in the present study was not accompanied by reported changes in mean corpuscular indices (MCV, MCH, MCHC), suggesting that fenugreek stimulated erythropoiesis while preserving normal erythrocyte morphology and hemoglobin content per cell, a pattern consistent with nutritional rather than pathological red cell responses [67]. Similarly, dietary supplementation with organic acids, particularly formic acid, significantly elevated RBC count, Hb concentration, and PCV values, which is consistent with previous studies in aquaculture species demonstrating that organic acids improve hematological status, erythropoiesis, nutrient utilization, and physiological health in fish [51,68].

The significant elevation in WBC count across supplemented groups, and particularly in the combined FA+FG group, reflects enhanced mobilization of leukocytes and activation of the non-specific cellular immune defense. Leukocyte proliferation is a hallmark response of the innate immune system to immunostimulatory dietary additives, and increased circulating WBC counts are widely associated with improved pathogen-clearing capacity in teleost fish [69,70]. The significant FA×FG interaction detected in both RBC and WBC by two-way ANOVA (*P* < 0.001) indicates that combined supplementation produced synergistic effects on hematological parameters, consistent with complementary mechanisms: FA improves mineral absorption and gut health, while FG's antioxidant phytochemicals protect blood cell integrity and stimulate hematopoiesis.

Serum total protein, albumin, and globulin concentrations serve as key indicators of nutritional status, hepatic synthetic function, and the competence of the humoral arm of the innate immune system in fish [71,72]. In the present study, all three parameters were significantly elevated in a rank order of G4 > G3 > G2 > G1, with the combined FA+FG treatment producing the highest values for all variables (*P* < 0.001). The elevation in serum total protein is primarily attributable to the enhanced dietary protein utilization associated with organic acid supplementation [22]. By lowering gastrointestinal pH, formic acid optimizes pepsin and protease activity, thereby improving the efficiency of protein hydrolysis and amino acid absorption, which, in turn, augments hepatic protein synthesis and plasma protein pools [25,73,74]. Additionally, the suppression of intestinal pathogenic bacteria by formic acid reduces competitive nutrient consumption and the energy cost of low-grade intestinal inflammation, channeling more absorbed amino acids toward anabolic processes and ultimately elevating circulating protein concentrations [59,75,76].

The significant increase in serum albumin concentration in supplemented groups reflects improved hepatic biosynthetic capacity and liver function, as albumin, synthesized exclusively by hepatocytes, is the predominant circulating protein responsible for maintaining oncotic pressure, transporting fatty acids, hormones, and minerals, and buffering plasma pH [22,77,78]. The enrichment of fenugreek diets with flavonoids and saponins likely contributed to this enhancement through their known hepatoprotective antioxidant activity, which mitigates lipid peroxidation in liver tissue and preserves hepatocellular structural and functional integrity, thereby supporting sustained albumin synthesis [[79], [80], [81]]. Consistent with these mechanistic inferences, several studies have demonstrated that dietary supplementation with organic acids or phytogenic additives increases serum albumin concentrations in teleost fish, reflecting improved liver function and protein metabolic efficiency [68,82,83].

The marked elevation in serum globulin, the most notable increase observed among the biochemical parameters, is a particularly significant finding from an immunological perspective. Globulins encompass the immunoglobulin fractions of plasma that constitute the primary humoral defense components in teleost fish, and elevated globulin concentrations are widely interpreted as indicative of an enhanced non-specific immune response and augmented capacity for antibody-like protein production [[84], [85], [86]]. The significant FA×FG interaction effects detected by two-way ANOVA for both albumin (*P* = 0.003) and globulin (*P* < 0.001) confirm that the combined treatment exerted synergistic effects on hepatic protein metabolism and humoral immune competence. This synergism is best explained by the complementary modes of action of the two additives: formic acid enhances the intestinal bioavailability of fenugreek's immunoactive phytochemicals by creating an optimal absorptive environment, while fenugreek's bioactive constituents directly stimulate hepatic metabolic activity and immune cell function, resulting in biochemical improvements that neither additive could achieve to the same magnitude independently [75,87].

Oxidative stress, resulting from an imbalance between reactive oxygen species (ROS) production and antioxidant defenses, is a major consequence of bacterial infection in fish and can lead to lipid peroxidation, cellular damage, immune dysfunction, and impaired physiological functions [88]. In the present study, dietary supplementation with FA, FG, or their combination significantly modulated the hepatic antioxidant response, as evidenced by elevated SOD and GPx activities and reduced MDA concentrations, with the greatest improvements observed in the combined treatment group (G4). The enzymatic antioxidants measured in this study function as key components of the cellular ROS-scavenging system, where SOD and GPx neutralize reactive oxygen species, while reduced MDA levels indicate lower lipid peroxidation and oxidative damage in supplemented fish [72,89].

Notably, the two-way ANOVA results revealed a nuanced pattern for SOD activity: while fenugreek supplementation exerted a significant main effect on SOD (*P* < 0.001), formic acid did not produce a significant main effect (*P* = 0.280), and no significant FA×FG interaction was detected (*P* = 0.567). Tukey post-hoc analysis confirmed that G1 and G2 were not significantly different from each other (*P* = 0.987), nor were G3 and G4 (*P* = 0.708), indicating that the SOD-elevating effect in this study was driven predominantly by fenugreek's phytochemical constituents rather than by formic acid per se [59,90]. In contrast, GPx activity and MDA concentration both showed significant FA×FG interactions (*P* = 0.004 and *P* < 0.001, respectively), indicating that the combined treatment produced synergistic improvements in these parameters. This differential pattern suggests that while fenugreek's polyphenols and flavonoids are the primary drivers of SOD upregulation, through their direct antioxidant radical-quenching activity and their capacity to induce antioxidant-responsive element (ARE)-mediated gene expression, formic acid contributes synergistically to the later enzymatic steps of the antioxidant cascade (GPx-mediated peroxide detoxification) and to the overall suppression of lipid peroxidation, likely through its role in improving gut barrier function and reducing ROS generation associated with intestinal dysbiosis and inflammation [28,91].

The significant FA×FG interaction for MDA is especially meaningful in the context of disease resistance, as oxidative stress is known to impair the functional activity of immune cells, reducing their phagocytic capacity, cytokine production, and bactericidal efficiency [27,36,90]. Therefore, the attenuated MDA concentrations observed in supplemented fish, and most markedly in the combined treatment group, likely contributed directly to the enhanced immune competence and improved post-challenge survival observed in these fish, establishing a mechanistic link between antioxidant status and disease resistance [30,38,92].

The innate immune system represents the first line of defense in teleost fish and plays a critical role in resistance against bacterial pathogens such as *A. hydrophila* [93,94]. In the present study, dietary supplementation with formic acid (FA), fenugreek (FG), and their combination significantly enhanced serum lysozyme activity and cytokine levels (IL-1β, TNF-α, and TGF-β), with the combined FA+FG group consistently exhibiting the strongest immune responses. Lysozyme is an essential bacteriolytic enzyme that hydrolyzes bacterial cell walls and contributes to antimicrobial defense against fish pathogens, including *A. hydrophila* [95,96]. The elevated lysozyme activity observed in supplemented groups indicates enhanced innate immune capacity, in agreement with previous studies reporting immunostimulatory effects of organic acids and phytogenic additives in fish [22,36,97,98]. Two-way ANOVA revealed significant effects of both FA and FG on lysozyme activity (*P* < 0.001), whereas the FA×FG interaction was non-significant (*P* = 0.629), suggesting additive rather than synergistic effects.

The significant increases in serum IL-1β and TNF-α levels, particularly in the combined FA+FG group, indicate activation of pro-inflammatory immune pathways involved in macrophage activation, leukocyte recruitment, phagocytosis, and bacterial clearance [38,99,100]. Significant FA×FG interactions for both cytokines (*P* < 0.001) further demonstrate synergistic stimulation of inflammatory immune responses by combined supplementation. In parallel, TGF-β levels were significantly elevated in supplemented fish, indicating activation of immunoregulatory pathways that help maintain immune balance and prevent excessive inflammatory damage [101,102]. The concurrent upregulation of pro-inflammatory mediators (*IL-1β* and *TNF-α*) alongside *TGF-β* reflects a regulated immune response in which early pro-inflammatory signaling facilitates pathogen clearance, while *TGF-β* simultaneously restrains excessive inflammation to limit collateral tissue damage, a pattern consistent with the regulatory cytokine axis described in teleost innate immunity [103,104]. Fenugreek phytochemicals likely enhanced cytokine signaling through NF-κB and MAPK pathways, whereas formic acid may have improved gut integrity and reduced basal inflammatory stress, thereby optimizing immune responsiveness [38,105].

Dietary FA and FG supplementation also significantly upregulated hepatic *IL-1β, TNF-α*, and *TGF-β* gene expression, with the combined FA+FG treatment producing the highest expression levels. These molecular findings support the protein-level immune responses and confirm enhanced transcriptional activation of immune pathways in supplemented fish. The increased expression of *IL-1β* and *TNF-α* following *A. hydrophila* challenge reflects activation of pro-inflammatory defense mechanisms essential for bacterial elimination [20,36,92]. Significant FA×FG interactions for *IL-1β* (P < 0.001) and *TNF-α* (*P* = 0.024) further indicate synergistic stimulation of cytokine gene expression by the combined treatment. In contrast, the FA×FG interaction for *TGF-β* expression was non-significant (*P* = 0.522), suggesting that fenugreek played the dominant role in regulating anti-inflammatory responses. Fenugreek polyphenols likely modulated immune signaling pathways such as NF-κB and JAK-STAT [106], while formic acid improved intestinal health and reduced inflammatory stress associated with microbial imbalance [28]. Moreover, the reduced oxidative stress observed in supplemented fish, evidenced by lower MDA levels, may have further supported efficient cytokine gene transcription and immune responsiveness.

The *A. hydrophila* challenge test confirmed the practical significance of these physiological, antioxidant, and immunological improvements. Fish fed FA- and FG-supplemented diets exhibited significantly higher survival rates than the control group, with the combined FA+FG treatment providing the greatest protection (96.7%), followed by FG alone (91.7%), FA alone (83.3%), and the control group (75.0%) (χ² = 14.135, P = 0.003). The improved disease resistance likely resulted from the integrated enhancement of innate immunity, antioxidant defense, and physiological status. Increased WBC counts and lysozyme activity indicate stronger first-line immune defense, while the upregulation of IL-1β and TNF-α at both protein and gene expression levels suggests enhanced inflammatory responses required for effective pathogen clearance. Concurrent elevation of TGF-β further indicates balanced immune regulation that may limit excessive inflammatory tissue damage. These findings are consistent with previous studies demonstrating the protective effects of formic acid [30,51,107] and fenugreek [36,38,47,98] supplementation against *A. hydrophila* infection in aquatic animals.

Enhanced antioxidant status, characterized by increased SOD and GPx activities and reduced MDA levels, also contributed to improved disease resistance by protecting immune cells against oxidative stress associated with bacterial infection and phagocytic activity. In addition, higher globulin concentrations in supplemented groups suggest improved humoral immune activity and enhanced production of immune-related proteins involved in pathogen neutralization and opsonization. The superior survival observed in the combined FA+FG group highlights the practical value of dual supplementation, in which complementary mechanisms of organic acids and phytogenic compounds provide stronger protection than either additive alone. The progressive improvement in survival (G1 < G2 < G3 < G4) closely paralleled the enhancement observed in growth, antioxidant, hematological, and immune parameters, supporting the mechanistic consistency of the present findings and agreeing with previous studies on multifunctional feed additives in aquaculture [108].

## Conclusions

The present study demonstrates that dietary supplementation with formic acid (0.5%) and fenugreek powder (2%), both individually and in combination, significantly enhances growth performance, hematological and biochemical parameters, antioxidant defense, innate immune function, and immune-related gene expression in Nile tilapia, culminating in markedly improved resistance to *A. hydrophila* infection. The combined FA+FG treatment consistently produced the greatest improvements across all measured parameters, with two-way ANOVA confirming significant FA×FG interactions for most growth, biochemical, and pro-inflammatory cytokine variables, indicating synergistic enhancement, while other markers (SOD activity, lysozyme activity, and TGF-β expression) responded additively, suggesting that FA and FG act through both shared and independent physiological pathways. These synergistic effects are attributable to the complementary mechanisms of action of the two supplements: formic acid optimizes the gastrointestinal environment, enhancing nutrient and phytochemical bioavailability and suppressing pathogenic bacterial colonization, while fenugreek's bioactive phytochemicals directly stimulate antioxidant enzyme expression, immune cell function, and cytokine-related gene transcription. Together, these findings support the combined dietary application of formic acid and fenugreek as an effective, safe, and sustainable alternative to prophylactic antibiotic use in Nile tilapia aquaculture. Future studies should investigate dose optimization for large-scale commercial systems, assess the long-term effects of prolonged supplementation on intestinal microbiome composition, and evaluate the economic feasibility of this dual-additive strategy under practical farming conditions.

## Ethical approval statement

All experimental procedures involving Nile tilapia (Oreochromis niloticus) were conducted in accordance with international guidelines for the care and use of laboratory animals, including EU Directive 2010/63/EU and the ARRIVE guidelines. The study protocol was reviewed and approved by the Institutional Animal Care and Use Committee (IACUC) of Al-Furat Al-Awsat Technical University under approval number Iq.AAU.REC.2026.024. All efforts were made to minimize animal suffering, reduce the number of fish used, and ensure the reliability and scientific validity of the experimental results.

## Funding

No funding.

## CRediT authorship contribution statement

**Hussein M.K. Al-Mohammad:** Writing – review & editing, Writing – original draft, Validation, Software, Resources, Methodology, Investigation, Formal analysis, Data curation, Conceptualization. **Dooha K. Alshamarey:** Writing – review & editing, Resources, Methodology. **Ahmed J. Obaid:** Writing – review & editing, Visualization, Methodology, Investigation, Formal analysis. **Mahmoud Alagawany:** Writing – review & editing, Software, Methodology, Formal analysis. **Mohammed F. El Basuini:** Writing – review & editing, Writing – original draft, Visualization, Validation, Software, Methodology, Investigation, Formal analysis, Data curation. **Akram Ismael Shehata:** Writing – review & editing, Writing – original draft, Visualization, Validation, Software, Methodology, Investigation, Formal analysis, Data curation.

## Declaration of competing interest

The authors declare that they have no known competing financial interests or personal relationships that could have appeared to influence the work reported in this paper.

## Data Availability

Data will be made available on request.
